# Genetic Variation and Hybridisation among Eight Species of kōwhai (*Sophora*: Fabaceae) from New Zealand Revealed by Microsatellite Markers

**DOI:** 10.3390/genes9020111

**Published:** 2018-02-20

**Authors:** Peter Heenan, Caroline Mitchell, Gary Houliston

**Affiliations:** 1 Research Associate, Allan Herbarium, Landcare Research, PO Box 40, Lincoln 7640, New Zealand; 2 Landcare Research, PO Box 40, Lincoln 7640, New Zealand; mitchellc@landcareresearch.co.nz (C.M.); houlistong@landcareresearch.co.nz (G.H.)

**Keywords:** Fabaceae, hybridisation, kōwhai, microsatellite markers, New Zealand, *Sophora* section *Edwardsia*, species concepts

## Abstract

We analysed nine microsatellite markers for 626 individuals representing the geographic range of eight closely related endemic New Zealand species of *Sophora*. Structure analysis identified the optimal *K* value as seven, with samples identified as *Sophora chathamica*, *Sophora fulvida*, *Sophora longicarinata*, and *Sophora prostrata* retrieved as well-defined groups. The remaining samples formed less resolved groups referable to *Sophora tetraptera* and *Sophora godleyi*, with *Sophora microphylla* and *Sophora molloyi* forming the seventh group. Our data suggest that considerable admixture occurs and this is most likely the result of hybridisation or introgression. *S. fulvida* shows admixture with the sympatric *S. chathamica*, and the widespread *S. microphylla* exhibits admixture with the sympatric *S. godleyi*, *S. molloyi*, and *S. tetraptera*.

## 1. Introduction

*Sophora* section *Edwardsia* represents a species radiation centred in the Pacific Ocean, with eight species in New Zealand, five species in Chile (including Juan Fernandez Islands, and Easter Island), and several species on various islands scattered across the Pacific Ocean (Lord Howe Island, Hawaii, and French Polynesia) [1,2]. Furthermore, the Chilean *Sophora cassioides* also occurs on Gough Island in the south Atlantic Ocean, and *Sophora denudata* is restricted to Réunion Island in the western Indian Ocean. The subtropical beach strand *Sophora tomentosa* is the type species for the genus *Sophora* and is sister to all species of sect. *Edwardsia* [3].

The New Zealand species of *Sophora*, collectively known by the indigenous Māori name kōwhai, have been the subject of recent taxonomic studies resulting in the recognition of eight endemic tree and shrub species [4,5,6]. Elucidating species relationships has proven difficult, with traditional phylogenetic markers (e.g., chloroplast DNA (cpDNA) *atp*B-*rbc*L spacer and *rbc*L, nuclear ribosomal DNA (nrDNA) internal transcribed spacer (ITS)) offering little sequence variation among the 19 species of *Sophora* sect. *Edwardsia* [1,3,7]. Other attempts to establish species relationships have unsuccessfully used enzyme electrophoresis [8] and amplified fragment length polymorphism (AFLP) markers [9]. Inter simple sequence repeats (ISSR) markers resolved *Sophora prostrata* samples as a group, but the samples of the other species did not form discrete species groups [9]; a similar result was obtained with more recently generated AFLP data [10]. Another recent study [11] used the same ISSR markers as [9] and reported that *S. prostrata* individuals were mixed up with individuals of *Sophora microphylla* and *Sophora tetraptera*.

A phylogeographic study [10] of 416 samples representing the eight New Zealand species using 22 chloroplast *trnQ-5′rps16* haplotypes concluded that little of the genetic variation was partitioned by species boundaries or geography, haplotype diversity decreased from North Island (20 haplotypes, nine haplotypes unique) to South Island (11 haplotypes, two haplotypes unique), and chloroplast sharing among the New Zealand species most probably results from hybridisation and introgression. A phylogenetic analysis of *Sophora* sect. *Edwardsia* [12] used cpDNA *trnQ-5′rps16* and *trnH*^GUG^*-psbA* intergenic spacers and the nuclear-encoded chloroplast-expressed glutamine synthetase gene and detected low genetic diversity. These authors showed that chloroplast haplotypes and nuclear alleles were shared across the southern Pacific Ocean between New Zealand (eight species) and Chile (including Juan Fernandez Islands) (*S. cassioides*, *Sophora macrocarpa*, and *Sophora fernandeziana*). In contrast, northern Pacific Ocean species have unique chloroplast haplotypes, including the individual species from Hawaii (*Sophora chrysophylla*), Easter Island (*Sophora toromiro*), and Lord Howe Island (*Sophora howinsula*). The three French Polynesia species (*Sophora raivavaeensis, Sophora rapaensis*, and *Sophora mangaraevaensis*) share the same haplotype, and *S. denudata* from Réunion Island in the Indian Ocean also has a unique haplotype.

The failure of the earlier genetic studies—e.g., [1,3,7,8,9,11]—to elucidate species relationships, and setting aside the more informative recent analyses [2,10,12], prompted the development of 12 microsatellite markers [13]. These microsatellite markers discriminated 14 individuals of *Sophora chathamica* and four populations of *S. microphylla* and have provided tools that will enable genetic variation among individual plants to be studied. Indeed, the markers have been successfully used to investigate the mating system and inbreeding depression among kōwhai populations [14].

In this study, we analyse genetic data obtained from nine microsatellites markers [13] and 626 individuals to test the circumscription of the eight New Zealand endemic *Sophora* species that are recognised on the basis of growth habit, leaf, and floral characters [4,5,6]. These data are shown to provide support for the recognition of most of the New Zealand species and also provide evidence of hybridisation and gene flow among the species.

## 2. Materials and Methods

### 2.1. Plant Material

The eight endemic New Zealand *Sophora* species were sampled for this study, totalling 626 individual samples. These are *S. chathamica* Cockayne (92 samples), *Sophora fulvida* (Allan) Heenan & de Lange (57 samples), *Sophora godleyi* Heenan & de Lange (55 samples), *Sophora longicarinata* G. Simpson & J.S. Thomson (27 samples), *S. microphylla* Aiton (149 samples), *Sophora molloyi* Heenan & de Lange (15 samples), *S. prostrata* Buchanan (115 samples), and *S. tetraptera* J.F. Mill. (116 samples). Herbarium vouchers for all samples included in the study are deposited in Allan Herbarium (CHR), Landcare Research, Lincoln, New Zealand (Table S1).

To test the reproducibility of the microsatellites, we included duplicates of 37 samples, usually represented by herbarium sheets labelled with a unique sheet number and the suffix A, B, or C. While it is not known with certainty that these samples were collected from the same plant, this is the usual practice when collecting and labelling duplicate herbarium collections.

### 2.2. Microsatellite Markers

The markers used were described by [13], who developed 12 microsatellite markers for *S. microphylla*. Nine out of the 12 polymorphic loci were used to genotype 626 samples in this study. Two primers (Sop-831 and Sop-834) were not used as they did not consistently amplify in the species included in this study, while Sop-807 was removed due to the presence of many null alleles.

Samples were extracted with a DNeasy Plant Mini Kit (Qiagen, Hilden, Germany) following the manufacturer’s protocol. DNA extractions were run through a Nanodrop ND-1000 spectrophotometer (Nanodrop Technologies, Wilmington, KY, USA) where the concentration ranged between 7–24 ng/uL. PCR was performed in 15 µL reactions in multiplexes with a maximum of three primer sets per multiplex. Reactions consisted of 1 µL DNA at 7–24 ng and final concentrations of 1x iNtRON i-Taq PCR buffer (iNtRON Biotechnology, Seongnam, South Korea), 208 µM iNtRON i-Taq dNTP mix (iNtRON Biotechnology), 0.1 µg/uL bovine serum albumin (New England Biolabs, Ipswich, MA, USA), forward primer labelled either 167 nM (Sop-445, Sop-42, Sop-814) 267 nM (Sop-808) 467 nM (Sop-802, Sop-816, Sop-806) or 533 nM (Sop-248, Sop-825), reverse primer at the same concentrations as per the forward, 0.5U iNtRON i-Taq polymerase (iNtRON Biotechnology). PCR conditions were as follows: initial denaturation of 94 °C for 5 min followed by 35 cycles of 94 °C for 30 s, 53 °C for 30 s, and 72 °C for 45 s, then an extension of 72 °C for 30 min. 1 uL from the PCR product was added to 9 uL Hi-Di formamide (Applied Biosystems, Waltham, MA, USA) and 1 uL LIZ-labelled size standard (Applied Biosystems) before being separated on an ABI 3130xl Genetic Analyser (Applied Biosystems) at the Landcare Research sequencing laboratory (Auckland, New Zealand). Alleles were visualised and scored using GeneMarker v2.6.0 (SoftGenetics, State College, PA, USA).

### 2.3. Genetic Analyses

Summary statistics were calculated in GenAlEx v6.501 [15] to determine for each species the total number of alleles, number of private alleles, observed heterozygosity and expected heterozygosity at each microsatellite loci for each species, with the percentage of missing data per loci for each species was noted. To determine the level of differentiation between populations, the pairwise fixation statistic (F_ST_) was calculated for each population in Arlequin v3.5.2.2 [16], and the presence of null alleles was checked with Microchecker v2.2.3 [17]. Structure v2.3.4 [18] was used to assess genetic structure among the species using the following parameters on the entire dataset: a run length of 10^6^ Markov chain Monte Carlo (MCMC) cycles following 10^5^ burn-in cycles, no admix model, correlated allele frequencies, *K* value 1–9, 10 independent runs (iterations) performed for each *K*. The optimal *K* value from the Structure output was determined from Δ*K*, and the rate of change in the log probability over all 10 iterations [19], calculated within Structure Harvester v0.6.94 [20]. To ensure all iterations from the optimal *K* value were consistent, the Structure results were run through Clumpak [21].

A genetic distance matrix was calculated on the entire dataset in GenAlEx v6.501 and used to draw a principal coordinates analysis (PCoA) in MVSP v3.1 [22]. The samples were mapped using ArcGIS v10.5 [23], with the colour of each sample corresponding to that which occurred on the structure plot with the optimal *K* value of seven. For samples with a mixed genotype, the inferred ancestry probabilities for each *K* from Structure were used and if a sample had greater than 0.5 probability for a particular *K*, and then that corresponding colour was chosen.

## 3. Results

Summary statistics for each species are reported in Table 1. The mean observed heterozygosity was lower than the expected heterozygosity across the majority of loci for each species. The mean observed heterozygosity ranged from 0.44 (*S. fulvida*) to 0.69 (*S. prostrata*), and the mean expected heterozygosity ranged from 0.60 (*S. fulvida*) to 0.80 (*S. prostrata*). *S. microphylla* had the highest number of alleles across all loci (148), while *S. molloyi* had the least (60). All species except *S. fulvida* had private alleles, with *S. prostrata* having the highest number (19). The private alleles were distributed across all nine loci. Missing data (no alleles scored at a locus) was observed at locus Sop-802 in *S. longicarinata* (25%), *S. prostrata* (16%), *S. tetraptera* (6%) and *S. microphylla* (6%) and at locus Sop-806 in *S. molloyi* (6%), and *S. prostrata* (8%). The average F_ST_ for most populations was low-moderate [24], at 0.051–0.103, but with *S. longicarinata* the highest at 0.146 and *S. microphylla* the lowest at 0.045, suggesting it is the least genetically distinct population. Null alleles were identified in each loci and spread across the populations, with Sop-802 having the highest frequency (0.206) and Sop-248 with the lowest (0.069). Across all loci and populations, the average null allele frequency was 0.12.

The reproducibility of the microsatellites was tested in seven of the eight species of *Sophora* and was confirmed by the majority of the duplicate samples being identical as follows: *S. chathamica* (seven samples duplicated, seven identical); *S. fulvida* (4/8); *S. godleyi* (5/5); *S. longicarinata* (2/2); *S. microphylla* (5/5); *S. prostrata* (5/5); *S. tetraptera* (5/5). Most of the duplicate samples were pure, but some were admixed, and these were also identical (e.g., *S. fulvida*, samples 122 and 123; *S. godleyi*, samples 167 and 168; *S. microphylla*, samples 466 and 467; *S. tetraptera*, samples 539 and 540). The mixed allelles observed in the four *S. fulvida* samples probably result from collections having been mixed in the field or when being processed in the herbarium.

**Figure 1:**
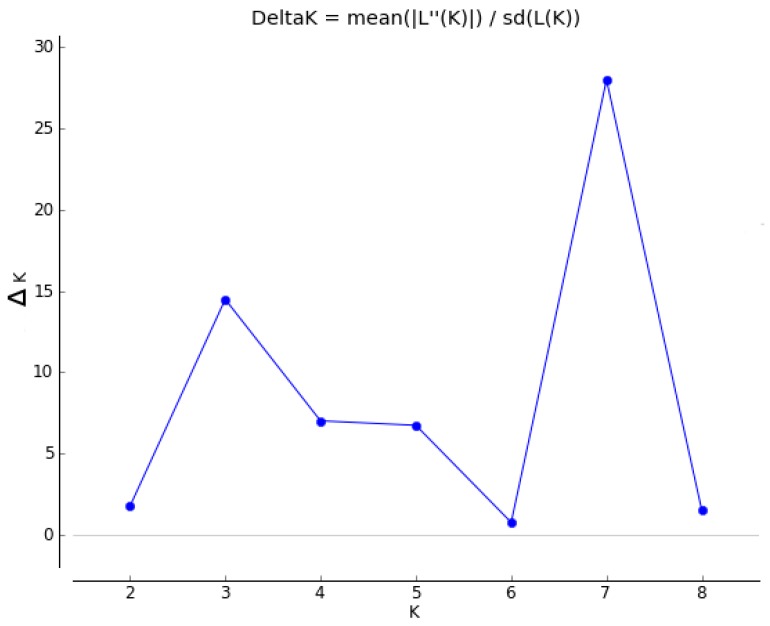
Δ*K* output from Structure Harvester.

**Figure 2:**
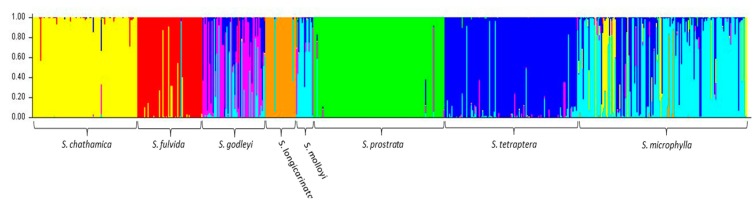
Output from Structure for the optimal value of *K* (*K*7). The colours refer to the genotype of each sample, with the samples being grouped by their morphological identification.

Analysis of the entire dataset using Structure and Structure Harvester suggested that the optimal *K* value was seven (Figure 1), with all 10 iterations being consistent as displayed in Clumpak. The Structure analysis assigned most individuals within a species to a single cluster with a high probability, with the exception of *S. godleyi,* which appeared to be made up of individuals that belong to several different clusters (Figure 2). Several samples in *S. microphylla* and *S. molloyi* also had lower assignment probabilities to a single cluster, as well as having significant similarity in assignment of individuals within these species. *Sophora fulvida, S. prostrata*, and *S. chathamica* were all well recovered in the Structure analysis, although there were some individuals that were assigned partially to both the *S. fulvida* and *S. chathamica* clusters. *S. tetraptera* and *S. longicarinata* also produced strongly defined clusters of individuals, with only a few assigned to other clusters within the analysis.

**Figure 3:**
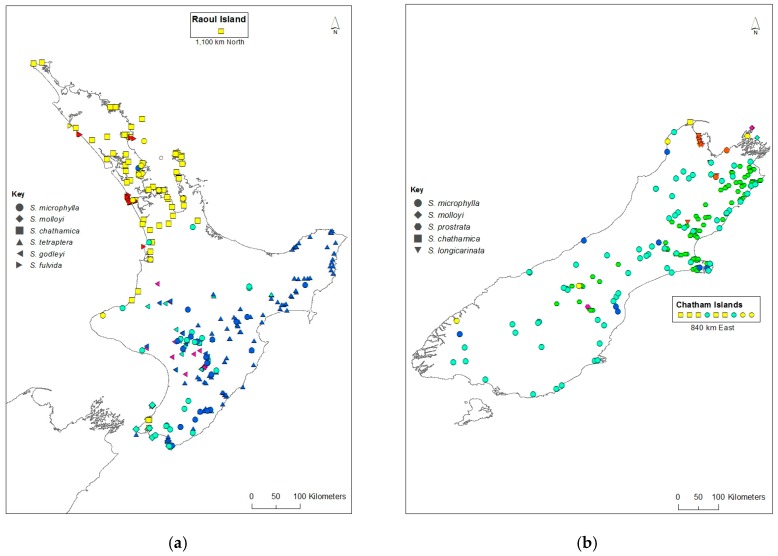
Distribution of *Sophora* species in New Zealand. (**a**) North Island (inset Raoul Island); (**b**) South Island (inset Chatham Islands). Each species is represented by a different shaped symbol. The symbol colours correspond with the colours in the Structure plot (see Figure 2).

**Figure 4:**
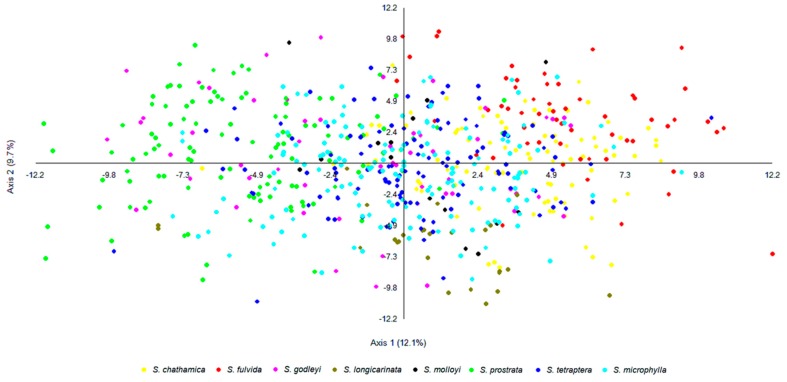
Genetic clustering based on a Principal Coordinates Analysis using the software MVSP [22]. The first two axes explain 12.1% and 9.7% of the total variance. The symbol colours correspond to the morphological groups (see Figure 2).

For samples that are admixed, the inferred ancestry probabilities are assigned to the allele with greater than 0.5 representation for a particular *K*. Each sample was assigned a single colour corresponding to the dominant assignment and this was mapped (Figure 3). There are both taxonomic and geographic patterns. *S. chathamica*, *S. fulvida*, *S. longicarinata*, and *S. prostrata* are each characterised by almost all samples being in a single cluster. On the other hand, *S. microphylla* is primarily assigned to a single cluster but also has some samples assigned to the clusters predominantly assigned to other species from throughout its distributional range. For example, in the lower North Island (Wairarapa region, NZ), samples of *S. microphylla* assign to the same cluster as the majority of *S. tetraptera*. *S. godleyi* and *S. molloyi* each have individuals assigned to three different clusters. The *S. molloyi* samples from the lower North Island mostly cluster with the majority of samples assigned to *S. microphylla* and *S. tetraptera*, and those from northern South Island are clustered with *S. godleyi* and *S. microphylla*. *S. tetraptera* is assigned mostly to one cluster but has a few samples assigned to the main *S. microphylla* group. Samples from the northern offshore Raoul Island (Kermadec Islands) are represented by *S. chathamica*, and those from the eastern Chatham Islands are shown to be *S chathamica* and *S. microphylla* (Figure 3) The PCoA (Figure 4) shows weak clustering among some of the species, and with axes 1 and 2 explaining 12.1% and 9.7% of the variation, respectively. On axis 1 the most distinctive clusters are provided by the *S. prostrata* samples grouped on the left-hand side and those of *S. chathamica* and *S. fulvida* on the right hand side. Most samples of *S. longicarinata* and *S. molloyi* each form groups in the central part of the PCoA.

## 4. Discussion

### 4.1. Structuring of Genetic Diversity and Taxonomic Concepts

In this study, we have used nine microsatellite markers to characterise the eight New Zealand *Sophora* species. Structure analyses of our data assigned many of the 626 samples to groups corresponding with seven of the eight New Zealand endemic species of *Sophora*, a result that is mostly consistent with the morphological identification of the samples. These results are a significant step forward in corroborating the species circumscriptions based on morphology and habitat specificity [4,6] and are in stark contrast to previous AFLP, ISSR, and enzyme electrophoresis studies [8,9,11] that did not provide genetic support for the species concepts.

Six of the eight species of *Sophora* included in this study were retrieved by Structure analysis (Figure 2). This is a significant result since, prior to the recent taxonomic revision of *Sophora* in New Zealand [4,6], *S. chathamica* was not an accepted species and *S. fulvida* and *S. longicarinata* were treated as varieties of *S. microphylla* [25]. In contrast, *S. prostrata*, with its divaricate growth habit, small and few leaflets, and resupinate flower, has long been accepted as a distinct species, and in previous genetic studies, it is one of the few species to have been distinguished [9,10], a result supported in this study. However, *S. molloyi* appeared to be similar in assignment to *S. microphylla*, and *S. godleyi* comprised individuals with a range of assignment values as well as samples that appeared to be uniquely assigned to *S. godleyi* (pink samples in Figure 2). The close relationship between *S. microphylla* and *S. molloyi* is a similar result to that of Shepherd et al. [10] who showed that in the southern North Island the two species share haplotype *K* and in the southern part of Cook Strait they share haplotype F. *Sophora microphylla* and *S. molloyi* are distinguished by their growth habit, leaf characters and ecological preferences as presented in the taxonomic revision [6].

A noteworthy feature of the genetic variation reported here is that it is comparable to the genetic diversity reported for the symbiotic *Mesorhizobium* root nodule bacteria that have an obligate relationship with New Zealand *Sophora* species. Based on phylogenetic analyses of the 16S ribosomal RNA (rRNA), *recA*, *glnll*, and *rpoB* genes [26] and DNA–DNA hybridisations, matrix-assisted laser desorption/ionization time-of-flight (MALDI-TOF) mass spectronomy (MS) analysis, enterobacterial repetitive intergenic consensus (ERIC)-PCR, physiological tests and phenotypic differentiation, seven *Mesorhizobium* species have been newly named and described [27,28]. Further sampling of *Sophora* root nodule bacteria has identified additional lineages that probably represent as yet unnamed species of *Mesorhizobium* [29]. A feature of these two genera is that they both show evidence of the movement of DNA between species: interspecific hybrids occur in *Sophora* as shown here and by [10], and horizontal gene transfer has been suggested to occur in *Mesorhizobium* [29]. Taken together, the *Sophora* and *Mesorhizobium* genetic data possibly infers parallel diversification in New Zealand. The drivers of this host and symbiont diversity are not known and require further study, as do the specifics of the host-symbiont-ecological relationships. Nevertheless, we suggest that abiotic characteristics may be important evolutionary drivers of both *Sophora* and *Mesorhizobium*. Species of both genera occur naturally on a range of parent rock types and soils (e.g., mudstone, volcanic outcrops, and limestone), and New Zealand has diverse and steep environmental gradients that influence species distributions [6,26], such as temperature varying with latitude and rainfall varying with longitude.

### 4.2. Hybridisation

In addition to most of the *Sophora* species being assigned to a single cluster, our data also comprises a smaller number of samples that appear to be admixtures, and these provide genetic evidence that the species hybridise. This result is consistent with a recent study [10], which showed that chloroplast haplotype variation is not partitioned by species boundaries and is most likely explained by hybridisation and introgression. Taken together, these two studies provide compelling genetic data supporting earlier accounts of hybrids based on morphology [6,25,30,31].

The Structure analysis provides two lines of genetic evidence that are indicative of the occurrence of interspecific hybridisation in *Sophora*. These are samples that exhibit admixture and where a sample has a genetic profile that differs from the common genetic profile that is typical for that species. *S. microphylla* has the greatest potential to form hybrids as it occurs throughout the main New Zealand islands, where it is sympatric with all of the other species whose distributions are more geographically restricted. There are several notable examples of possible hybrids involving *S. microphylla*. First, the majority of the samples of *S. microphylla* assigned to the *S. chathamica* cluster (yellow in Figure 2 and Figure 3) come from North Auckland or Taranaki, areas where *S. microphylla* and *S. chathamica* often grow naturally together. In addition, there are two plants from Chatham Island that were identified in the field as *S. microphylla* [32], but these have been placed with the *S. chathamica* group. These two plants are characteristic of *S. microphylla* in having a divaricating juvenile growth form and the leaves having distantly placed and uniformly sized leaflets. These two plants were collected from a limestone outcrop on the western edge of Te Whanga Lagoon, where they are syntopic with *S. chathamica*. The origin of these plants cannot be known, but they may be the result of introgressive hybridisation and are expressing the morphology of *S. microphylla* and the genetic profile of *S. chathamica*. Two other plants (e.g., CHR 529950), grown from seed collected from the beach strand line on Chatham Island, have the genetic profile of *S. microphylla*. The second example relates to *S. longicarinata*, which has two samples with the genetic profile of *S. longicarinata* but the morphology of *S. microphylla*. One of these samples comes from Cable Bay (Nelson) where *S. longicarinata* is not known, and the other comes from Leatham River valley, where *S. longicarinata* occurs [4]. Two other samples from a population growing on limestone at the Ure (Waima) River in Marlborough are identified as *S. longicarinata* but have the genetic profile of *S. microphylla*. These two samples lack a divaricating growth habit and have the leaf characters of *S. longicarinata*, and typical divaricating *S. microphylla* occurs nearby in the same catchment.

A notable example of possible reciprocal interspecific hybrids is provided by *S. chathamica* and *S. fulvida*, two species that are sympatric in northern New Zealand (Figure 3). Figure 2 shows three admixed samples of *S. chathamica* with a portion of the genetic profile comprising the signature of *S. fulvida*, and the reciprocal situation occurs with eight samples of *S. fulvida* being admixed with *S. chathamica*. These eight samples occur in the Waitakere Ranges and at Maunganui Bluff (northern North Island), localities where both species have been observed to occur (P.B. Heenan pers. obs.).

*S. godleyi* exhibits considerable genetic variation (Figure 2 and Figure 3), which is an interesting result since all 55 samples used for the genetic analyses are morphologically consistent with that species. *S. microphylla* is sympatric with *S. godleyi* throughout the range of the latter species, and *S. tetraptera* is known from the eastern part of the range of *S. godleyi*; hybrids between *S. godleyi* and these two species were previously documented [6]. A preliminary analysis in NewHybrids [33] (data not shown) of the *S. godleyi* samples showed the class assignment with ≥95% probability placed most samples in the categories of F2, backcross, or a hybrid of not just one type (data not shown). The pink genotype displayed in the *K*7 Structure plot (Figure 2) comprised mostly samples that were identified as either parent A or a backcross to parent A, and these probably represent “pure” *S. godleyi*. However, the high proportion of hybrids and backcrosses and weak parental assignment of samples suggests there is poor support for separating these samples into different groups. Therefore, the results should be treated with caution, as NewHybrids may not perform well with distinguishing between the different classes when there is weak genetic differentiation and low number of markers [34]. 

In this study, we treated each species as a discrete population since our individual samples are collected from different sites and therefore are not large enough to allow useful comparison at the population level. Comparison of heterozygosity for each species (Table 1) shows that observed heterozygosity (H_O_) is lower than expected heterozygosity (H_E_). This could partly be attributed to the null alleles that occur across the dataset. However, this is an expected result since random mating (panmixis) is not likely to occur across the widely sampled range of each species. In contrast, it is to be expected that in a population comprising a single species or when two species grow in close proximity there will be an increased opportunity for random mating and also interspecific hybridisation. Breeding systems have an obvious influence on intraspecific gene flow and the formation of interspecific hybrids. The New Zealand species of *Sophora*, as exemplified by *S. microphylla* [35], are self-compatible and have flowers with a classic ornithophilous syndrome of yellow petals and abundant nectar. These flowers are considered to be adapted to pollination by the New Zealand endemic honeyeaters tui (*Prosthemadera novaezelandiae*) and the bellbird (*Anthornis melanura*) of the family Meliphagidae [36,37]. Tui and bellbird will almost certainly move pollen between different *Sophora* species when they occur in close proximity, and since there are no apparent mating restrictions or obvious reproductive isolating mechanisms, this could result in fertilisation and the formation of seeds. This would account for the wild hybrids reported here and that have been previously documented [6,10,25,30,31]. Inbreeding depression has been reported [35], in which *S. microphylla* had high high fruit set after cross-pollination but significantly reduced fruit set after selfing, and the selfed plants exhibited inbreeding depression. Thus, although *Sophora* has a mixed mating system and can produce both selfed and outcrossed seed, the true recruits in a population are only the outcrossed fruits and seedlings [35]. This breeding system favouring outcrossing is also likely to facilitate interspecific hybridisation as much as outcrossing among conspecifics. It has previously been argued [6] that in a natural ecosystem each species’ edaphic and habitat requirements are rather specific and natural habitats that could be suitable for hybrid plants have perhaps been uncommon. These authors observed that many hybrid plants occurred in sites that have been disturbed through land clearance and the incursion of weeds, and that the hybrid plants could be opportunistic in occupying these novel habitats. Opportunities for hybridisation could be limited by differences in phenology between some of the species [6].

The genetic data presented here does not provide analyses at the population level or at specific sites where two or more species may occur together along with putative hybrids. Hybridisation can be simple (e.g., F1 hybrids only) or complex (e.g., with F2 hybrids, backcrosses, or hybrid swarms). Often, stable hybrid zones can occur when there is habitat differentiation or when there is no habitat differentiation then either one of the parental species or the hybrids can displace the other taxa [38]. With the latter scenario, it may take as few as five generations for hybridisation to replace one of the parent species. Since this study and the recent phylogeographic study [10] have both detected *Sophora* hybrids, further field-based studies with targeted sampling of species that are sympatric are required to elucidate the types and extent of hybridisation that occurs in natural populations.

The presence of putative *Sophora* hybrids creates a potential problem of assigning a name to individual plants. This is particularly so when the genetic profile of the sample is inconsistent with its morphological characteristics, and this is indicative of hybridisation or introgression. We suggest that when a sample has the genetic profile of one species or is admixed, but has the morphological characters of another species, these plants should be treated pragmatically and referred to the species whose morphology they are characterised by. However, some samples are probably better treated as hybrids, particularly when both their morphology and genetic admixture is indicative of a hybrid origin.

## Figures and Tables

**Table 1:** Genetic diversity parameters for eight *Sophora* species from nine microsatellite markers.

Locus	*S. chathamica* (*n* = 92)			*S. fulvida* (*n* = 57)			*S. godleyi* (*n* = 55)			*S. longicarinata* (*n* = 27)			*S. molloyi* (*n* = 15)			*S. prostrata* (*n* = 115)			*S. tetraptera* (*n* = 116)			*S. microphylla* (*n* = 149)		
	*A*	*H_o_*	*H_e_*	*A*	*H_o_*	*H_e_*	*A*	*H_o_*	*H_e_*	*A*	*H_o_*	*H_e_*	*A*	*H_o_*	*H_e_*	*A*	*H_o_*	*H_e_*	*A*	*H_o_*	*H_e_*	*A*	*H_o_*	*H_e_*
Sop-445	7	0.29	0.37	5	0.23	0.29	5	0.31	0.60	4	0.19	0.21	4	0.27	0.56	7	0.67	0.77	7	0.53	0.53	10	0.55	0.67
Sop-802	17	0.66	0.88	12	0.45	0.80	16	0.56	0.80	13	0.40	0.87	9	0.13	0.82	12	0.50	0.83	21	0.60	0.92	20	0.71	0.91
Sop-816	9	0.46	0.66	4	0.37	0.54	11	0.72	0.78	6	0.70	0.74	5	0.60	0.68	8	0.60	0.66	12	0.67	0.73	10	0.59	0.76
Sop-248	21	0.78	0.90	15	0.78	0.90	21	0.70	0.91	16	0.81	0.90	11	0.80	0.88	19	0.89	0.92	20	0.79	0.90	23	0.85	0.92
Sop-42	8	0.42	0.55	5	0.21	0.26	7	0.53	0.62	7	0.56	0.53	4	0.40	0.50	13	0.52	0.64	12	0.58	0.69	15	0.54	0.64
Sop-806	14	0.61	0.76	9	0.37	0.69	16	0.49	0.84	8	0.67	0.71	7	0.57	0.81	30	0.69	0.92	16	0.72	0.76	21	0.73	0.82
Sop-808	14	0.45	0.56	12	0.58	0.76	20	0.55	0.73	10	0.81	0.73	8	0.67	0.70	30	0.85	0.92	22	0.67	0.77	26	0.64	0.75
Sop-814	4	0.52	0.66	3	0.44	0.50	6	0.33	0.71	6	0.37	0.61	4	0.27	0.70	5	0.63	0.74	6	0.64	0.73	7	0.55	0.74
Sop-825	10	0.67	0.86	8	0.56	0.66	11	0.71	0.86	11	0.74	0.88	8	0.60	0.83	11	0.83	0.83	11	0.77	0.88	16	0.76	0.88
Mean	11.6	0.54	0.69	8.1	0.44	0.60	12.6	0.54	0.76	9.0	0.58	0.69	6.7	0.48	0.72	15.0	0.69	0.80	14.1	0.66	0.77	16.4	0.66	0.79
*A_T_*	104			73			113			81			60			135			127			148		
*A_P_*	3			0			8			1			1			19			8			9		

Note: *n* = number of individuals sampled; *A* = number of alleles; *H_o_* = observed heterozygosity; *H_e_* = expected heterozygosity; *A_T_* = total number of alleles; *A_P_* = number of private alleles.

## Supplementary Materials

The following are available online at http://www.mdpi.com/2073-4425/9/2/111/s1, Table S1: Samples used for the study.
